# Endophytic fungal association via gibberellins and indole acetic acid can improve plant growth under abiotic stress: an example of *Paecilomyces formosus *LHL10

**DOI:** 10.1186/1471-2180-12-3

**Published:** 2012-01-12

**Authors:** Abdul Latif Khan, Muhammad Hamayun, Sang-Mo Kang, Yoon-Ha Kim, Hee-Young Jung, Joong-Hwan Lee, In-Jung Lee

**Affiliations:** 1 School of Applied Biosciences, Kyungpook National University, Daegu, Republic of Korea; 2 Department of Plant Sciences, Kohat University of Science & Technology, Kohat Pakistan; 3 Department of Botany, Abdul Wali Khan University, Mardan Pakistan; 4 Division of Applied Biology and Chemistry, College of Agriculture and Life Sciences, Kyungpook National University, Daegu, Republic of Korea; 5 Gyeongsangbuk- Do Agricultural Research and Extension Services, Division of Agriculture Environment Research, Daegu, Republic of Korea

**Keywords:** *Paecilomyces formosus *LHL10, Salinity, Cucumber plant growth, Gibberellins and indole acetic acid, Endogenous plant hormones

## Abstract

**Background:**

Endophytic fungi are little known for exogenous secretion of phytohormones and mitigation of salinity stress, which is a major limiting factor for agriculture production worldwide. Current study was designed to isolate phytohormone producing endophytic fungus from the roots of cucumber plant and identify its role in plant growth and stress tolerance under saline conditions.

**Results:**

We isolated nine endophytic fungi from the roots of cucumber plant and screened their culture filtrates (CF) on gibberellins (GAs) deficient mutant rice cultivar *Waito-C *and normal GAs biosynthesis rice cultivar Dongjin-byeo. The CF of a fungal isolate CSH-6H significantly increased the growth of *Waito-C *and Dongjin-byeo seedlings as compared to control. Analysis of the CF showed presence of GAs (GA_1_, GA_3_, GA_4_, GA_8_, GA_9_, GA_12_, GA_20 _and GA_24_) and indole acetic acid. The endophyte CSH-6H was identified as a strain of *Paecilomyces formosus *LHL10 on the basis of phylogenetic analysis of ITS sequence similarity. Under salinity stress, *P. formosus *inoculation significantly enhanced cucumber shoot length and allied growth characteristics as compared to non-inoculated control plants. The hypha of *P. formosus *was also observed in the cortical and pericycle regions of the host-plant roots and was successfully re-isolated using PCR techniques. *P. formosus *association counteracted the adverse effects of salinity by accumulating proline and antioxidants and maintaining plant water potential. Thus the electrolytic leakage and membrane damage to the cucumber plants was reduced in the association of endophyte. Reduced content of stress responsive abscisic acid suggest lesser stress convened to endophyte-associated plants. On contrary, elevated endogenous GAs (GA_3_, GA_4_, GA_12 _and GA_20_) contents in endophyte-associated cucumber plants evidenced salinity stress modulation.

**Conclusion:**

The results reveal that mutualistic interactions of phytohormones secreting endophytic fungi can ameliorate host plant growth and alleviate adverse effects of salt stress. Such fungal strain could be used for further field trials to improve agricultural productivity under saline conditions.

## Background

Various crops cultivated in arid or semi-arid regions are frequently exposed to wide range of environmental stresses. Among these, salinity severely affects plant growth and metabolism and hence results in reduced biomass production. Plants have the capability to cope with these stresses through many signal transduction pathways adjusting their metabolism [1-3]. These adjustments range from changes in ionic/osmotic levels, stomatal closure to changes in phytohormones and secondary metabolites [4]. Sodium ion toxicity trigger the formation of reactive oxygen species (ROS) such as superoxide (O_2_^-^), hydrogen peroxide (H_2_O_2_), and hydroxyl radical (•OH) which can ultimately damage; (i) mitochondria and chloroplasts, (ii) water use efficiency, (iii) photosynthesis, and (iv) nutrients uptake whilst disrupting cellular structures [1,4]. To avoid oxidative damage, plants adapt by *de novo *synthesis of organic compatible solutes acting as osmolytes. Osmolytes like proline serve a free-radical scavenger stabilize subcellular structures and buffer cellular redox potential under stress [5]. In counteracting oxidative stress antioxidant molecules are also involved as defence strategy.

Symbioses with beneficial fungi can ameliorate plant growth and its physiological status [6]. Endophytic fungi comprise of fungal symbionts associated with plants living inside tissues without causing any disease symptoms [7-11]. Endophytes have mostly been reported for their behaviour to enhance plant growth as they influence key aspects of plant physiology and host protection against biotic and abiotic stresses [9,10,12]. Besides that, endophytic fungi have been known as an important source of various kinds of bioactive secondary metabolites [8,13]. It has been known recently that some of the strains of endophytic fungi can produce plant hormones especially gibberellins (GAs) [14]. Under extreme environmental conditions, these phytohormone producing endophytic fungi can effect the production of several secondary metabolites like flavonoids [15] along with phytohormones to help the plant to tolerate/avoid stress [8,12,16]. GAs are ubiquitous substances that elicit various metabolic functions required during plants' growth [17,18]. However, little is known about GAs production by endophytic fungi and their role in abiotic stress. Previously, various strains of fungal species including endophytes have been reported to either secrete GAs in their culture medium or have an active GAs biosynthesis pathway. Fungal species like *Gibberella fujikuroi*, *Sphaceloma manihoticola *[18], *Phaeosphaeria *sp., *Neurospora crassa *[19], *Sesamum indicum *[20], *Phaeosphaeria *sp. L487 [21], *Penicillium citrinum *[14], *Chrysosporium pseudomerdarium *[22] and *Scolecobasidium tshawytschae *[23], *Aspergillus fumigatus *[15] and *Penicillium funiculosum *[16] have been reported as GAs producers. GAs along with other plant hormones like indole acetic acid (IAA) secreted by fungal endophytes can improve plant growth and crop productivity [24,25].

Aim of the present study was to identify plant hormone (GAs and IAA) secreting endophytic fungal strain and assess its role in host-plant physiology under saline conditions. For this purpose, isolated endophytic fungal strains were initially screened on GAs deficient mutant rice cultivar (*Waito-C*) and GAs cultivar (Dongjin-byeo) seedlings to differentiate between plant growth promoting/inhibiting and plant hormones producing strain. The best fungal strain identified was examined for its potential role in plant growth under sodium chloride (NaCl) induced salinity stress. To elucidate the mitigation of oxidative stress imposed by NaCl, photosynthesis rate, stomatal conductance, transpiration rate, relative water content (RWC), electrolytic leakage (EL), free proline content, nitrogen assimilation, antioxidant and lipid peroxidation were analyzed. Endogenous ABA and GAs (GA_3_, GA_4_, GA_12 _and GA_20_) were quantified to understand the influence of salt stress and endophytic fungal association on the growth of cucumber plant.

## Materials and methods

### Endophyte isolation and screening

We collected 120 roots pieces from the field grown cucumber plants (four). Root pieces were surface sterilized with 2.5% sodium hypochlorite (30 min in shaking incubator at 120 rpm) and washed with autoclaved distilled water (DDW) to remove the contaminants, rhizobacteria and mycorrhizal fungi. The root pieces (0.5 cm) were carefully placed in petri-plates containing Hagem media (0.5% glucose, 0.05% KH_2_PO_4_, 0.05% MgSO_4_.7H_2_O, 0.05% NH_4_Cl, 0.1% FeCl_3_, 80 ppm streptomycin and 1.5% agar; pH 5.6 ± 0.2). The sterilized roots were also imprinted on separate Hagem plates to ensure the effectiveness of surface sterilization [14]. Endophytic fungi were isolated according to the method described by Khan et al [14] and Hamayun et al. [22,23]. The newly emerged fungal spots from the roots were isolated and grown on potatodextrose agar (PDA) medium under sterilized conditions [14]. Total nine different fungal strains were isolated and grown on PDA media. These strains were inoculated in Czapek broth (50 ml; 1% glucose, 1% peptone, 0.05% KCl, 0.05% MgSO_4_.7H_2_O, and 0.001% FeSO_4_.7H_2_O; pH 7.3 ± 0.2) and grown for seven days (shaking incubator -120 rpm; temperature 30°C) to separate liquid culture medium and fungal mycelia (centrifugation 2500*x*g at 4°C for 15 min). The culture medium (culture filtrate-CF, 50 ml) and mycelium (5.4 gm) were immediately shifted to -70°C freezer and then freeze-dried (Virtis Freeze Dryer, Gardiner, NY, USA) for 4-7 days. The lyophilized CF was diluted with one ml of autoclaved DDW, while the mycelia were used for genomic DNA extraction.

Presence or absence of plant growth promoting metabolites in fungal CF was confirmed by performing screening bioassays on gibberellins biosynthesis deficient mutant rice *Waito-C *and normal GAs cultivar *Oryza sativa *L. cv. Dongjin-byeo. *Waito-C *has dwarf phenotype while Dongjin-byeo has normal phenotype. For bioassay experiment, rice seeds were surface sterilized with 2.5% sodium hypochlorite for 30 minutes, rinsed with autoclaved DDW and then incubated for 24 hr with 20-ppm uniconazol (except Dongjin-byeo) to obtained equally germinated seeds. Then pre-germinated *Waito-C *and Dongjin-byeo seeds were transferred to pots having water: agar medium (0.8% w/v) [14] under aseptic conditions. Both the rice cultivars were grown in growth chamber (day/night cycle: 14 hr- 28°C ± 0.3;10 hr - 25°C ± 0.3; relative humidity 70%; 18 plants per treatment) for ten days. Ten micro-litter of fungal CF was applied at the apex of the rice seedlings. One week after treatment, the shoot length, chlorophyll content and shoot fresh weight were recorded and compared with negative (autoclaved DDW) and positive controls (*Gibberella fujikuroi*). The wild-type strain of *G. fujikuroi *KCCM12329, provided by the Korean Culture Center of Microorganisms, was used as positive control. Upon screening results, bioactive fungal strain CSH-6H was selected for further experiments and identification.

### Fungal DNA isolation, identification and phylogenetic analysis

Genomic DNA was extracted from CSH-6H using standard method of Khan et al. [14]. Fungal isolate was identified by sequencing the internal transcribed region (ITS) of rDNA using universal primers: ITS-1; 5'-TCC GTA GGT GAA CCT GCG G-3' and ITS-4; 5'-TCC TCC GCT TAT TGA TAT GC-3'. The BLAST search program (http://blast.ncbi.nlm.nih.gov) was used to compare the nucleotide sequence similarity of ITS region of related fungi. The closely related sequences obtained were aligned through CLUSTAL W using MEGA version 4.0 software [26] and a maximum parsimony tree was constructed using the same software. The bootstrap replications (1K) were used as a statistical support for the nodes in the phylogenetic tree.

### Endophytic interactions and stress application

Experiments were conducted with a completely randomized block design in order to assess the endophytic fungus relationship with host-plants. Experiments comprised of cucumber (*Cucumis sativus *L) plants with (i) fungal inoculation, (ii) without inoculation, (iii) fungal inoculation with salt stress (60 and 120 mM), and (iv) without inoculation and salt stress. On the basis of results obtained in *Waito-C *and Dongjin-byeo screening bioassay, the bioactive endophytic fungal strain (CSH-6H) was inoculated in Czapek broth (250 ml) as described in endophyte isolation and screening section. Similarly, cucumber seeds before sowing in autoclaved pots were surface sterilized as described earlier. The germinated seeds (28°C and relative humidity of 60%) were grown in autoclaved pots (200 g/pot of soil at 121°C for 90 min). The fungal mycelia and culture filtrate (20 ml for each pot containing ten propagules) were added to substrate composed of peat moss (13-18%), perlite (7-11%), coco-peat (63-68%) and zeolite (6-8%), with macro-nutrients present as: NH_4_- ~90 mg Kg^-1^; NO_3_- ~205 mg Kg^-1^; P_2_O5 ~350 mg Kg^-1 ^and K_2_O ~100 mg Kg^-1 ^[12-14]. The control plants only received 20 ml/pot of endophyte-free medium (containing 1% glucose, 1% peptone, 0.05% KCl, 0.05% MgSO_4_.7H_2_O, and 0.001% FeSO_4_.7H_2_O; pH 7.3 ± 0.2; shaking for 10 days at 30°C). The endophytic fungi and cucumber plants were grown together for three weeks in growth chamber (day/night cycle: 14 hr- 28°C ± 0.3;10 hr - 25°C ± 0.3; relative humidity 60-65%; 18 plants per treatment) and irrigated with distilled water. After three weeks, NaCl solution (300 ml/plant) was applied to cucumber plants for one week in order to assess the affect of salt stress on these plants.

The growth parameters i.e. shoot length, shoot fresh and dry weights were measured for harvested cucumber plants, while chlorophyll content of fully expanded leaves were analyzed with the help of chlorophyll meter (SPAD-502 Minolta, Japan). Dry weights were measured after drying the plants at 70°C for 72 h in oven. Total leaf area was measured with Laser Leaf Area meter (CI-203 model, CID Inc., USA). Portable photosynthesis measurement system (ADC BioScientific LCi Analyser Serial No. 31655, UK) was used to calculate the net photosynthetic rate (μmolm^-2^s^-1^), transpiration rate (mMm^-2^s^-1^) and stomatal conductance (molm^-2^s^-1^) per unit leaf area of fully expanded leaves. For each measurement, readings were recorded in triplicates. For endogenous phytohormonal analysis of cucumber plants, the treated samples were immediately frozen in liquid nitrogen and kept until further use at -70°C. Samples were freezed dried in Virtis Freeze Dryer (Gardiner, NY, USA).

### Microscopic analysis

Cucumber roots inoculated with CSH-6H were sectioned and treated with sodium hypochlorite (2.5%) for 10 min for clarification. Experimental conditions were kept aseptic during analysis. Inoculated roots were treated with 20% KOH for 24 h and rinsed with autoclaved DDW. The roots were then acidified with 10% HCl, stained overnight using 0.05% 0.1% acid fuchsin and 95% lactic acid. Finally, the roots were destained in 95% lactic acid for 24 h. The roots pieces were then subjected to light microscope (Stemi SV 11 Apo, Carl Zeiss). The root parts having active colonization were used for re-isolation of the inoculated CSH-6H with the method as described earlier.

### RWC, EL, proline, nitrogen assimilation, antioxidant and lipid peroxidation

Relative water content (RWC) and electrolytic leakage (EL) were measured following González and González-Vilar [27]. Free proline was estimated following Bates et al. [28]. Plant samples were oven-dried at 65°C and were ground to pass through 1-mm mesh sieves and analyzed for N using CNS analyzer (Carlo-Erba NA1500, Carlo Erba Instruments, Milano, Italy). Antioxidant activity was measured on the basis of radical scavenging activity of 1, 1-diphenyl-2-picrylhydrazyl (DPPH) as described Xie et al. [29]. The extent of lipid peroxidation was determined by the method of Ohkawa et al. [30]. The experiments were repeated three times.

### GAs extraction from fungal CF and cucumber plants

To characterize GAs secreted in the pure fungal culture of bioactive endophyte, it was inoculated in Czapek broth (120 ml) for 7 days at 30°C (shaking incubator-120 rpm) as described previously [14,24]. The culture and mycelium were separated by centrifugation (2500*x*g at 4°C for 15 min). The culture medium (CF; 50 ml) was used to extract and purify GAs as described by Hamayun et al. [22,23]. Briefly, the pH of the CF was adjusted to 2.5 using 6 N HCl and was partitioned with ethyl acetate (EtOAc). Before partitioning, deuterated GAs internal standards (20 ng; [17, 17-^2^H_2_] GA_1_, GA_3_, GA_4_, GA_8_, GA_12 _and GA_24_) were added in the CF. Tritiated GAs i.e. [1, 2-^3^H_2_] GA_9 _and [1,2-^3^H_2_] GA_20 _were also added (obtained from Prof. Lewis N. Mander, Australian National University, Canberra, Australia). The organic layer was vacuum dried and added with 60% methanol (MeOH) while the pH was adjusted to 8.0 ± 0.3 using 2 N NH_4_OH. Similarly, endogenous GAs from cucumber plants treated with and without endophytic fungus and salinity stress were extracted from 0.5 g of freeze-dried plant samples according to the method of Lee et al. [31]. About 20 ng each of deuterated [17, 17-^2^H_2_] GA_3_, GA_4_, GA_12 _and GA_20 _internal standards were added. The CF and plant extracts were subjected to chromatographic and mass spectroscopy techniques for identification and quantification of GAs.

### Chromatography and GC/MS - SIM for hormonal analysis

The extracts were passed through a Davisil C18 column (90-130 μm; Alltech, Deerfield, IL, USA). The eluent was reduced to near dryness at 40°C in vacuum. The sample was then dried onto celite and then loaded onto SiO_2 _partitioning column (deactivated with 20% water) to separate the GAs as a group from more polar impurities. GAs were eluted with 80 ml of 95: 5 (v ⁄ v) ethyl acetate (EtOAc): hexane saturated with formic acid. This solution was dried at 40°C in vacuum, re-dissolved in 4 ml of EtOAc, and partitioned three times against 4 ml of 0.1 M phosphate buffer (pH 8.0). Drop-wise addition of 2 N NaOH was required during the first partitioning to neutralize residual formic acid. One-gram polyvinylpolypyrrolidone (PVPP) was added to the combined aqueous phases, and this mixture was slurried for 1 h. The pH was reduced to 2.5 with 6N HCl. The extract was partitioned three times against equal volumes of EtOAc. The combined EtOAc fraction was dried in vacuum, and the residue was dissolved in 3 ml of 100% MeOH. This solution was dried on a Savant Automatic Environmental Speedvac (AES 2000, Madrid, Spain). The dried samples were subjected to high performance liquid chromatography (HPLC) using a 3.9 × 300 m Bondapak C18 column (Waters Corp., Milford, MA, USA) and eluted at 1.0 ml/min with the following gradient: 0 to 5 min, isocratic 28% MeOH in 1% aqueous acetic acid; 5 to 35 min, linear gradient from 28% to 86% MeOH; 35 to 36 min, 86% to 100% MeOH; 36 to 40 min, isocratic 100% MeOH. Forty-eight fractions of 1.0 ml each were collected (Additional file 1). The fractions were then prepared for gas chromatography/mass spectrometry (GC/MS) with selected ion monitoring (SIM) system (6890N Network GC System, and 5973 Network Mass Selective Detector; Agilent Technologies, Palo Alto, CA, USA). For each GAs, 1 μl of sample was injected in GC/MS SIM (Additional file 2). Full-scan mode (the first trial) and three major ions of the supplemented [17-^2^H_2_] GAs internal standards and the fungal GAs were monitored simultaneously whereas the same was done for endogenous GAs of cucumber plants (Supplementary data 2). The fungal CF GAs (GA_1_, GA_3_, GA_4_, GA_8_, GA_9_, GA_12_, GA_20 _and GA_24_) and the endogenous cucumber plant's GAs (GA_3_, GA_4 _and GA_12_) were calculated from the peak area ratios of sample GAs to corresponding internal standards. The retention time was determined using hydrocarbon standards to calculate the KRI (Kovats retention index) value (Additional file 1). The limit of detection was determined for all GAs. GC/MS SIM limit of detection was 20 pg/ml for fungal CF and plant samples. The data was calculated in nano-grams per millilitre (for fungal CF) or nano-grams per grams fresh weight (for cucumber plants) while the analyses were repeated three times.

### IAA analysis

Samples were analysed with a High Performance Liquid Chromatograph (HPLC) system, equipped with a differential ultraviolet (UV) detector absorbing at 280 nm and a C18 (5 μm; 25 × 0.46 cm) column. Mobile phase was methanol and water (80:20 [v/v]) at a flow rate of 1.5 ml/min. The sample injection volume was 10 μl. Retention times for the analyte peaks were compared to those of authentic internal standards added to the medium and extracted by the same procedures used with fungal cultures. Quantification was done by comparison of peak area [32].

### Endogenous ABA analysis

The endogenous ABA was extracted according to the method of Qi et al. [33]. The extracts were dried and methylated by adding diazomethane. Analyses were done using a GC-MS SIM (6890N network GC system, and 5973 network mass selective detector; Agilent Technologies, Palo Alto, CA, USA). For quantification, the Lab-Base (ThermoQuset, Manchester, UK) data system software was used to monitor responses to ions of m/z 162 and 190 for Me-ABA and 166 and 194 for Me-[^2^H_6_]-ABA (supplementary data 2).

### Statistical analysis

The analysis of variance and multiple mean comparisons were carried out on the data using Graph Pad Prism software (version 5.0, San Diego, California USA). The purpose of these tests was to identify statistically significant effects and interactions among various test and control treatments. The significant differences among the mean values of various treatments were determined using Duncan's multiple range tests (DMRT) at *95% CI *using Statistic Analysis System (SAS 9.1).

## Results

### Effect of fungal CF on *Waito-C *and Dongjin-byeo rice growth

We isolated 31 endophytic fungi from 120 roots of cucumber plants suggesting an abundance level of 3.87 endophytes per root sample. These fungi were grown on Hagem media plates for seven days. The pure culture plates were grouped on the basis of colony shape, height and colour of aerial hyphae, base colour, growth rate, margin characteristics, surface texture and depth of growth into medium [34]. The morphological trait analysis reveals that only nine endophytes were different. The CF of these nine different endophytes were assayed on *Waito-C *and Dongjin-byeo rice seedlings to differentiate between growth stimulatory or inhibitory and plant hormones producing strains. The growth attributes of dwarf *Waito-C *(GAs mutant dwarf cultivar) and Dongjin-byeo (normal GAs cultivar) rice seedlings were recorded after a week of treatment and the data is given in Table 1 and Table 2. The results showed that CF application of CSH-6H to *Waito-C *and Dongjin-byeo rice seedlings exhibit significant growth promotion as compared to the CF of *G. fujikuroi *and DDW applied control rice seedlings. Endophyte, CSH-6H significantly increased the shoot growth of dwarf *Waito-C *rice in comparison controls. The CSH-6H applied CF exhibited higher chlorophyll content and shoot fresh weight of rice seedlings than controls (Table 1). A similar growth stimulatory trend of CSH-6H was observed on the Dongjin-byeo rice seedling with active GAs biosynthesis pathway and normal phenotype (Table 2). In other growth promoting strain, CSH-7C and CSH-7B improved the shoot growth, fresh weight and chlorophyll content of *Waito-C *and Dongjin-byeo rice seedlings but it was not significantly different than the CF of *G. fujikuroi *(Table 1 and Table 2). In growth suppressive strains, CSH-1A inhibited the growth of *Waito-C *and Dongjin-byeo as compared other endophytic fungal strains. Upon significant growth promoting results of CSH-6H, it was selected for identification and further investigation.

**Table 1:** Effect of CF of endophytic fungal strains isolated from the roots of field grown cucumber plants on the growth of *Waito-C *rice seedlings

Isolates	Shoot length (cm)	Fresh weight (g)	Chlorophyll contents (SPAD)
Control (Gf)	8.0 ± 0.18b	0.6 ± 0.03b	31.5 ± 0.39b
Control (DW)	6.1 ± 0.11d	0.5 ± 0.06c	29.9 ± 0.16c
CSH-1A	6.6 ± 0.11d	0.2 ± 0.05e	30.1 ± 0.24c
CSH-3C	7.2 ± 0.12c	0.3 ± 0.05d	31.1 ± 1.43b
**CSH-6H**	**9.8 ± 0.19a**	**0.9 ± 0.05a**	**32.9 ± 0.13a**
CSH-6D	7.3 ± 0.13c	0.4 ± 0.01d	29.3 ± 0.23c
CSH-7C	8.7 ± 0.12b	0.7 ± 0.03b	31.6 ± 0.31b
CSH-5C	8.4 ± 0.12b	0.5 ± 0.05c	31 ± 1.52b
CSH-7B	8.5 ± 0.16b	0.6 ± 0.07b	24.3 ± 1.22d
CSH-5D	8.3 ± 0.20b	0.6 ± 0.07b	31 ± 0.54b
CSH-8D	8.4 ± 0.13b	0.4 ± 0.02d	29.6 ± 0.77c

Control (Gf) = rice seedlings treated with the CF of a wild-type strain of *Gibberella fujikuroi *KCCM12329; Control (DW) = rice seedlings treated with autoclaved distilled water. SPAD = Soil plant analysis development. In each column, treatment means having different letter are significantly (*P *< 0.05) different as evaluated by DMRT. Values in the table refer to mean ± SD (*n *= 18).

**Table 2:** Effect of CF of endophytic fungal strains on the growth of *Oryza sativa *L. cv. Dongjin-beyo rice seedlings

Isolates	Shoot length (cm)	Fresh weight (g)	Chlorophyll contents (SPAD)
Control (Gf)	13.4 ± 0.41b	0.8 ± 0.04b	29.5 ± 0.40b
Control (DW)	10.0 ± 0.42d	0.6 ± 0.06c	20.0 ± 0.62d
CSH-1A	8.7 ± 1.44e	0.5 ± 0.05d	24.3 ± 1.21c
CSH-3C	11.3 ± 0.91c	0.6 ± 0.05c	20.0 ± 0.92d
**CSH-6H**	**15.6 ± 0.27a**	**1.1 ± 0.05a**	**31.8 ± 0.21a**
CSH-6D	10.6 ± 0.92c	0.4 ± 0.01d	29.3 ± 0.68b
CSH-7C	13.9 ± 1.0b	0.8 ± 0.08b	14.8 ± 0.71e
CSH-5C	10.0 ± 0.44d	0.5 ± 0.05d	15.3 ± 0.93e
CSH-7B	14.8 ± 0.57b	0.8 ± 0.07b	16.9 ± 2.71e
CSH-5D	13.3 ± 0.75b	0.9 ± 0.07b	23.0 ± 0.54c
CSH-8D	13.2 ± 0.41b	0.8 ± 0.02b	29.6 ± 0.73b

Control (Gf) = rice seedlings treated with the CF of a wild-type strain of *Gibberella fujikuroi *KCCM12329; Control (DW) = rice seedlings treated with autoclaved distilled water; SPAD = Soil plant analysis development. In each column, treatment means having different letter(s) are significantly (*P *< 0.05) different as determined by DMRT. Values in the table refer to mean ± SD (*n *= 18).

### Identification and phylogenetic analysis of bioactive endophyte

**Figure 1:**
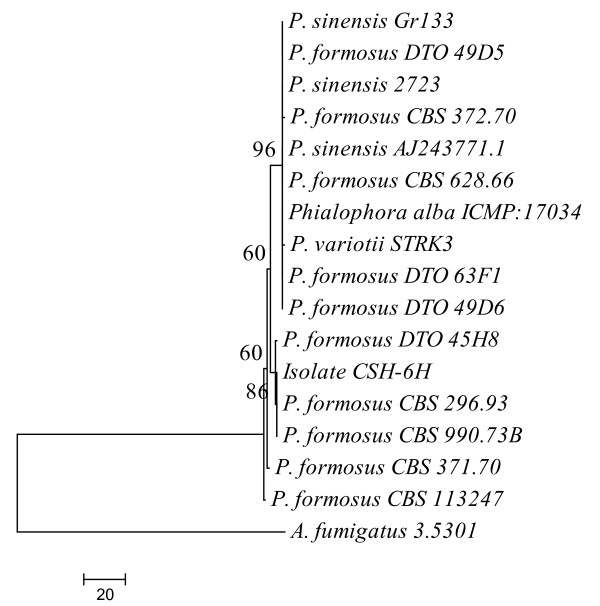
**Phylogenetic tree constructed through maximum parsimony method using MEGA 4.0 (Tamura et al. 2007)**. The sequence obtained from ITS regions of rDNA of *Paecilomyces formosus *LHL10 and related fungi. The bioactive endophytic fungal strain formed a sub-clade (86% bootstrap support) with *Paecilomyces *sp. *Aspergillus fumigatus *was taken as an out-group.

After DNA extraction and PCR analysis of ITS regions, phylogenetic analysis of CSH-6H was carried out [14,22,23]. Maximum parsimony (MP) consensus tree was constructed from 16 (15 references and 1 clone) aligned partial ITS regions sequences with 1 K bootstrap replications. Selected strains were run through BLAST search. Results of BLAST search revealed that fungal strain CSH-6H has 100% sequence similarity with *Paecilomyces sp*. In MP dendrogram CSH-6H formed 86% bootstrap support with *Paecilomyces formosus *(Figure 1). The sequence was submitted to NCBI GenBank and was given accession no. HQ444388. On the basis of sequence similarity and phylogenetic analysis results, CSH-6H was identified as a strain of *P. formosus *LHL10.

### Bioactive endophytic fungal CF analysis for phytohormones

**Figure 2:**
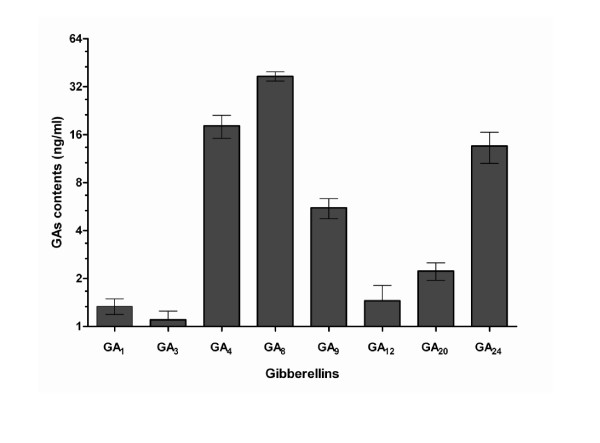
**Quantities of various GAs found in the CF of *P. formosus***. The experiment was repeated three times using already established method of Lee et al. (1998) through GC/MS-SIM. Each value is the mean ± SE of three replicates.

The CF of bioactive *P. formosus *(CSH-6H) was analysed for its potential to produce GAs in the growing medium. We detected 8 different physiologically active and non-active gibberellins (Figure 2) using GC/MS selected ion monitor. Among biologically active GAs, GA_1 _(1.3 ng/ml), GA_3 _(1.1 ng/ml) and GA_4 _(18.2 ng/ml) were found in the various HPLC fractions (Additional file 1). Among physiologically in-active GAs, GA_8 _(37.2 ng/ml), GA_9 _(5.5 ng/ml), GA_12 _(1.4 ng/ml), GA_20 _(2.2 ng/ml) and GA_24 _(13.6 ng/ml) were present in the CF. The quantities of bioactive GA_4 _and GA_8 _were significantly higher than the other GAs. Besides GAs, we also found IAA in the growing culture medium of *P. formosus*. The quantity of IAA was 10.2 ± 1.21 μg/ml.

### Effect of *P. formosus *association on cucumber growth in salinity stress

**Figure 3:**
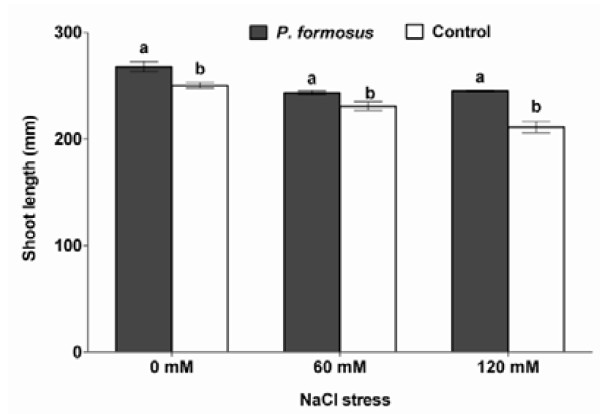
**Effects of NaCl induced salinity stress (0, 60 and 120 mM) on the shoot length of cucumber plants with or without endophytic interaction (*P. formosus*)**. Each value is the mean ± SE of 18 replicates per treatments. Different letter indicates significant (*P *< 0.05) differences between *P. formosus *inoculated plants and non-inoculated control plant as evaluated by DMRT.

**Figure 4:**
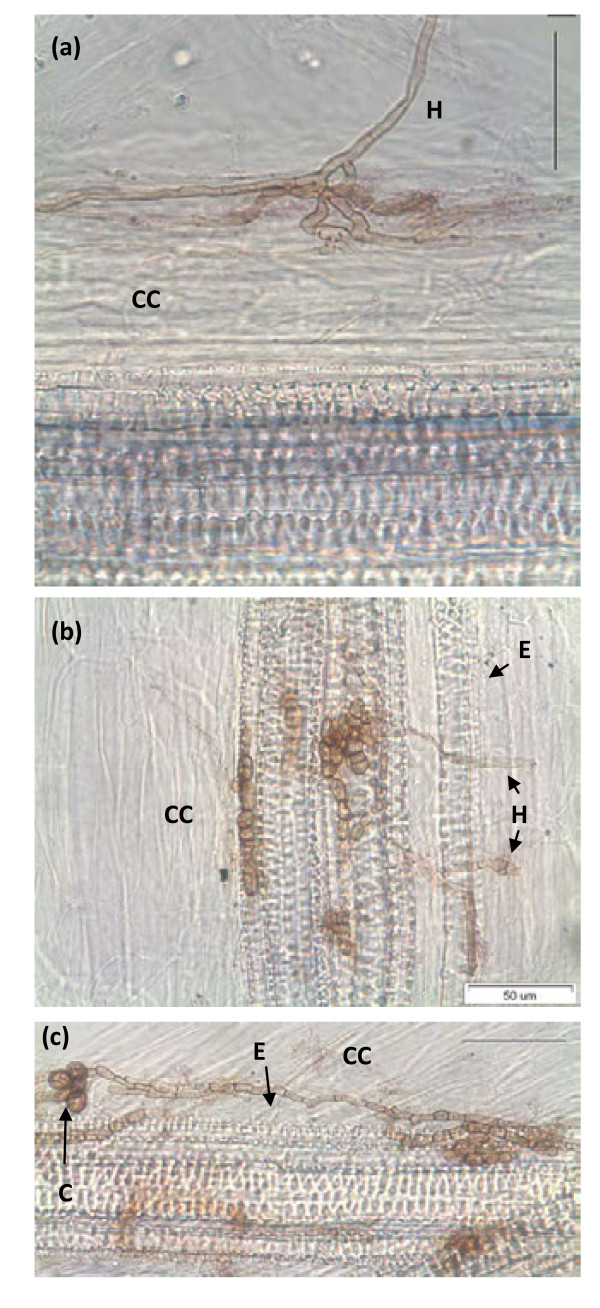
**Light microscopic analysis of cucumber root colonized by *P. formosus***. The fungus was observed: (a) forming hypha from epidermal region into cortical region; (b) developing in endodermal cells (c) switching to yeast-like cells or conidia in the periclycle region by undergoing morphological changes. H = Hypha; CC = cortex cells; E = endodermal cells; C = conidia or yeast like cells; scale bar 50 μm.

To assess the role of *P. formosus *in cucumber plant growth under saline soil condition, the endophyte was inoculated to the host plants. After three weeks of endophyte and host-plant association, NaCl was applied to induce salinity stress. The results reveal that the phytohormone producing *P. formosus *significantly increased the host-plant growth under normal growth conditions. The endophyte symbiosis increased the shoot length up to 6.89% as compared to non-inoculated control plants (Figure 3). Upon salinity stress of 60 mM, the plants inoculated with *P. formosus *had 4.5% higher shoot growth as compared to non-inoculated control. When exposed to 120 mM NaCl, endophyte-inoculated plants had 15.9% higher shoot length than control plants. *P. formosus *inoculated enhanced the chlorophyll content, shoot fresh and dry weights, photosynthesis rate, stomatal conductance and transpirational rate both under salinity stress in comparison to the non-inoculated control plants (Table 3). The light microscopic analysis also showed the active association and habitation of *P. formosus *inside the plant's root (Figure 4abc). Fungal hypha (brownish) has been observed in the cucumber plant roots (Figure 4a). The hypha from the epidermal region into cortex cells forms a dense network at the end in the cortex cells. The *P. formosus *was also observed in the endodermal cells occupying the pericycle region (Figure 4b). In the periclycle region, hyphae underwent further morphological changes, switching to yeast-like cells or conidia (Figure 4c). The fungus was re-isolated successfully from salinity stressed plants and was again identified through sequencing the ITS regions and phylogenetic analysis as mentioned earlier. Thus, confirming that *P. formosus *is responsible for establishing ameliorative interaction with host plants during stress conditions.

**Table 3:** Effect of salt stress on the growth of cucumber plants with or without endophyte inoculation

Growth attributes/salt stress	0 mM		60 mM		120 mM	
	
	Control	*P. formosus*	Control	*P. formosus*	Control	*P. formosus*
Chlorophyll content (SPAD)	27.3 ± 0.18b	29.1 ± 0.12a	28.0 ± 0.24b	36.5 ± 0.25a	24.3 ± 0.26b	37.1 ± 0.14a
Shoot fresh weight (g)	14.9 ± 0.33b	17.4 ± 0.15a	16.3 ± 0.29b	17.3 ± 0.16a	13.4 ± 0.35b	15.0 ± 0.41a
Shoot dry weight (g)	2.7 ± 0.07b	3.1 ± 0.08a	1.3 ± 0.01b	1.7 ± 0.02a	1.1 ± 0.01b	1.5 ± 0.09a
Leaf area (cm^2^)	58.6 ± 0.61b	62.1 ± 0.43a	48.9 ± 0.42b	52.4 ± 0.66a	40.9 ± 0.67b	43.1 ± 0.12a
Photosynthesis rate (μmolm^-2^s^-1^)	1.4 ± 0.05b	1.7 ± 0.02a	1.1 ± 0.03b	1.5 ± 0.04a	1.0 ± 0.06b	1.2 ± 0.03a
Stomatal conductance (molm^-2^s^-1^)	1.5 ± 0.02b	2.9 ± 0.01a	1.7 ± 0.06b	2.0 ± 0.03a	2.1 ± 0.02b	2.5 ± 0.08a
Transpiration rate (mMm^-2^s^-1^)	0.07 ± 0.01b	0.12 ± 0.01a	0.06 ± 0.01b	0.16 ± 0.01a	0.02 ± 0.01b	0.18 ± 0.01a

0 mM means only distilled water applied plants while 60 and 120 mM is the NaCl concentrations applied to the cucumber plants. SPAD = Soil plant analysis development. In each row, different letter indicates significant (*P *< 0.05) differences between *P. formosus *inoculated plants with non-inoculated control plant as evaluated by DMRT. Values in the table refer to mean ± SD (*n *= 18).

### Plant water potential and stress mitigation

**Figure 5:**
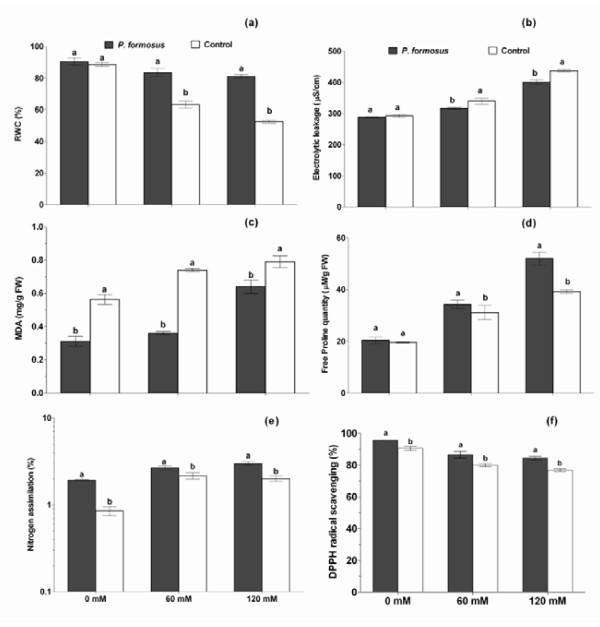
**Effects of the NaCl stress (0, 60 and 120 mM) on the relative water contents (a), electrolytic leakage (b), MDA content (c), free proline quantity (d), nitrogen assimilation (e), and antioxidant activity (f) of cucumber plants with or without endophytic inoculation (*P. formosus*)**. Each value is the mean ± SE of 3 replicates per treatments. Different letter indicates significant (*P *< 0.05) differences between *P. formosus *inoculated plants and non-inoculated control plant as evaluated by DMRT.

Relative water potential was not significantly different in *P. formosus *inoculated plants and non-inoculated plants. Under salinity stress (60 and 120 mM), the endophyte-inoculated cucumber plants showed significantly higher water potential as compared to the non-inoculated control plants (Figure 5a). The higher RWC indicates the beneficial endophytic association and rescuing role of *P. formosus *to curtail the adverse effects salinity stress. The electrolytic leakage (EL) from the cellular apparatus was almost similar in both endophyte associated plants and endophyte-free plants. However, upon salinity stress (60 and 120 mM), the non-inoculated control plants released significantly higher electrolytes as compared to *P. formosus *associated plants (Figure 5b). It suggests that the endophyte interaction counteracted the adverse effect of salinity by reducing the damage to the cellular membranes of the plants. The mitigating response of *P. formosus *association in salinity stress was further assessed the extent of lipid peroxidation. The results showed that MDA content was significantly lower in endophyte associated plants than control without NaCl stress. Upon salinity stress (60 and 120 mM), we again observed the significantly reduced levels of lipid peroxidation product (MDA) in the endophyte-inoculated plants than the control plants (Figure 5c).

The results showed that free proline quantity was not significantly different in cucumber plants inoculated with *P. formosus *and control. Treating cucumber plants with 60 mM NaCl stress, *P. formosus *inoculated plants had higher proline quantity in comparison to control. Cucumber plants when treated with 60 or 120 mM NaCl stress, *P. formosus *inoculated plants had higher proline quantity in comparison to controls (Figure 5d). To maintain the a high capacity of nitrogen for plant growth during salinity stress, we observed significantly higher assimilation of nitrogen endophyte-associated plant than endophyte free control plants with (60 and 120 mM) or without salinity stress (Figure 5e). Besides that, *P. formosus *inoculated plants exhibited higher oxidant radical scavenging by producing higher antioxidants as compared to control plants. After 60 and 120 mM NaCl application, the level of antioxidant production was significantly higher in *P. formosus *treated plants in comparison to non-inoculated control plants (Figure 5f).

### Effect of *P. formosus *on endogenous ABA and GAs under stress

**Figure 6:**
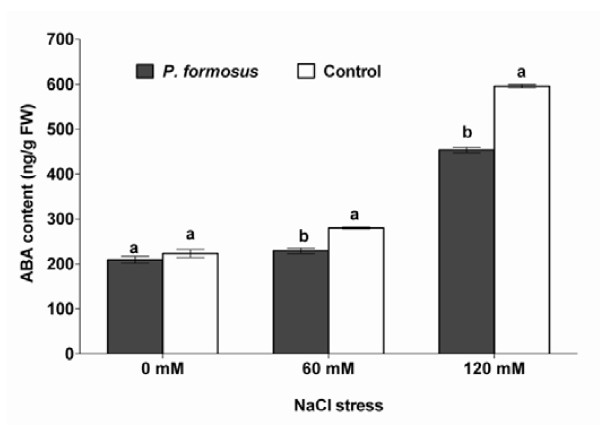
**Effect of NaCl induced salt stress on endogenous ABA content of the cucumber plants in the presence of *P. formosus *inoculation**. Each value is the mean ± SE of 3 replicates per treatments. Different letter indicates significant (*P *< 0.05) differences between *P. formosus *inoculated plants and non-inoculated control plant as evaluated by DMRT.

**Figure 7:**
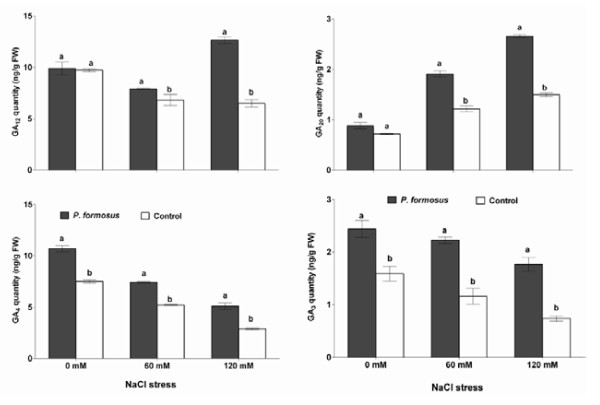
**Influence of salinity stress on the GAs (GA_3_, GA_4 _GA_12 _and GA_20_) contents of the plant's leaves with or without *P. formosus *inoculation**. Each value is the mean ± SE of 3 replicates per treatments. Different letter indicates significant (*P *< 0.05) differences between *P. formosus *inoculated plants and non-inoculated control plant as evaluated by DMRT.

Our results showed that the stress responsive endogenous ABA content in fungi inoculated plants was not significantly different than control plants. Upon NaCl stress treatments (60 and 120 mM) the cucumber plants with *P. formosus *association had significantly lower level of ABA content as compared to control plants (Figure 6). In case of endogenous GAs content, we analyzed the GA_12_, GA_20_, GA_4 _and GA_3 _of cucumber plants treated with or without salinity stress and *P. formosus*. We found that GA_12 _synthesis is almost same in both endophyte-associated and control plants under normal growth conditions. However, upon salinity stress (60 and 120 mM), the GA_12 _was significantly increased in endophyte-associated plants than the endophyte-free control plants (Figure 7). Similarly, GA_20 _was not significantly different in endophyte inoculated plants and control plants. After NaCl treatments (60 and 120 mM), the GA_20 _synthesis by cucumber plants inoculated with endophyte was significantly higher as compared to control plants (Figure 7). The GA_4 _content was significantly up-regulated in *P. formosus *associated plants than the control plants under normal and salinity stress (60 and 120 mM) conditions. A similar trend was also observed for GA_3 _contents (Figure 7).

## Discussion

We used screening bioassays and hormonal analysis of endophytic fungal CF in order to identify bioactive fungal strains, because fungi has been an exploratory source of a wide range of bioactive secondary metabolites [8,25]. In screening bioassays, rice cultivars were used as rice can easily grow under controlled and sterilized conditions using autoclaved water-agar media. *Waito-C *and Dongjin-byeo rice seedlings grown in hydroponic medium can help in assessment of CF obtained from endophytic fungi [14]. *Waito-C *is a dwarf rice cultivar with mutated *dy *gene that controls the 3β-hydroxylation of GA_20 _to GA_1_. The *Waito-C *seeds were also treated with GAs biosynthesis inhibitor (uniconazol) to further suppress the GAs biosynthesis mechanism [35]. Dongjin-byeo, on the other hand, has normal phenotype with active GAs biosynthesis pathway [35]. Since *Waito-C *and Dongjin-byeo growth media were devoid of nutrients, therefore, the sole effect of CF on rice was easily determined. Current study confirmed earlier reports stating that rice shoot growth stimulation or suppression can be attributed to the activity of plant growth promoting or inhibiting secondary metabolites present in the fungal CF [22,23]. The effect of CF from *P. formosus *was similar to that of *G. fujikuroi*, which possess an active GAs biosynthesis pathway [18]. *Waito-C *and Dongjin-byeo growth promotion triggered by the CF of *P. formosus *was later rectified as it contained physiologically active GAs and IAA. Upon significant growth promotive results in comparison to other fungal isolates, *P. formosus *was selected for identification and further investigation.

The endophytes releasing plant growth hormones, in present case, GAs and IAA can enhance plant growth. In current study, detection of GAs in the growing medium of *P. formosus *suggests that during interaction GAs were secreted causing growth promotion and also conferred ameliorative capacity to cucumber plants under salinity stress. Previous reports also confirm that fungal endophytes produce phytohormones. For instance, Hassan [24] reported that *Aspergillus flavus, A. niger, Fusarium oxysporum, Penicillium corylophilum, P. cyclopium, P. funiculosum *and *Rhizopus stolonifer *have the capacity to produce GAs, while *F. oxysporum *can secrete both GAs and IAA. Similarly, Khan et al. [16] reported that *P. funiculosum *can produce bioactive GAs and IAA. *Phaeosphaeria *sp. L487 was also found to possess GAs biosynthesis apparatus and can produce GA_1 _[21]. The CF of our fungal isolate also contained IAA, which is a molecule synthesized by plants and a few microbes [32], and has been known for its active role in plant growth regulation [36], while its biosynthesis pathway has been elucidated in bacterial strain [37]. The presence of IAA in *P. formosus *clearly suggests the existence of IAA biosynthesis pathway as reported for some other classes of fungi by Tuomi et al. [38]. Plants treated with endophytes are often healthier than those lacking such interaction [7-14], which may be attributed to the endophyte secretion of phytohormones such as IAA [16,36] and GAs [14-16,18,21-24]. In endophyte-host symbioses, secondary metabolites may be a contribution of the endophytic partner for such mutualistic relationship [9].

Endophytic fungi residing in root tissues and secreting plant growth regulating compounds are of great interest to enhance crop yield and quality. Such growth regulating compounds can influence plant development as well as rescue plant growth in stressful environments. Like many other plants, cucumber is more susceptible to salt stress [39,40]. Current study showed that *P. formosus *inoculation significantly improved plant growth and alleviated salinity induced stress. The presence of IAA and GAs in the CF of the fungus further rectifies our results, as both of them promote plant growth and development [41]. The presence of *P. formosus *in the cortical cells and their successful re-isolation by us further strengthens the active role of *P. formosus *in the host cucumber plants. The mutualistic relations of *P. formosus *with cucumber plant may have helped the host plant to mitigate the adverse effects of salinity stress. Similarly, recently Redman et al. [42] reported that IAA producing endophytic fungi can enhance rice plant growth under salinity, drought and temperature stress. Previously, Khan et al. [15,16] confirmed that GAs producing endophytic fungal strains (*P. funiculosum *and *Aspergillus fumigatus*) can ameliorate soybean plant growth under moderate and high salinity stress. Hamayun et al. [22,23] also reported that GAs secreting fungal endophytes promote soybean growth components. Many other studies also reported similar findings narrating that fungal interaction can enhance plants growth under stress conditions [9,12,43,44].

Plant growth and development depend upon leaf water contents, as salt stress trigger water deficit inside the plant tissues [4], and measurement of RWC helps to indicate stress responses of plant and relative cellular volumes [27]. Our current findings confirm earlier studies [43,44], suggesting that the fungal inoculated plants not only avoid stress but also help the plant to fetch higher water contents from sources usually inaccessible to control plants. Abiotic stresses cause higher electrolyte discharge (like K^+ ^ions) through displacement of membrane-associated Ca from plasma lemma. Resultantly, cellular membrane stability is damaged and aggregating higher efflux of electrolytes inside the plant tissues [27]. Our findings showed that plants associated with *P. formosus *had lower electrolytic leakage than control plants under salt stress. This indicated a lower permeability of plasma membrane attributed to the integrity and stability of cellular tissues due to endophyte-plant interaction as compared to control treatments [45]. On the other hand, antioxidant scavengers can enhance membrane thermostability against ROS attack, while MDA content can be used to assess injuries to plants [45]. It has been shown that peroxides of polyunsaturated fatty acids generate MDA on decomposition, and in many cases MDA is the most abundant individual aldehydic lipid breakdown product [30]. The higher MDA level is perceived with higher ROS production and cellular membrane damage. In our study, low levels of lipid peroxidation in *P. formosus *treated plants showed reduced cellular damage to cucumber plants growing under salinity stress as compared to control. Similarly, in saline conditions, osmo-protectants like proline accumulate to provide an energy source for plant growth and survival by preventing ionic and osmotic imbalances [46]. We observed significant aggregation of proline in *P. formosus *associated plants growing under salinity stress, suggesting a decline in ionic influx inside the cellular masses and rescuing cucumber plants to maintain its osmotic balance. Similarly, higher nitrogen uptake by endophyte-inoculated plants under salinity suggested the regulation of sodium ion toxicity to indirectly maintain chlorophyll and osmotic balance [47]. Sodium and chloride ion toxicity can trigger the formation of ROS which can damage cellular functioning [45-48]. Resultantly, accumulation of antioxidants inside plant can extend greater resistance to oxidative damage [48]. Higher DPPH radical scavenging activity in *P. formosus *inoculated plants suggest greater oxidative stress regulation than non-inoculated plants [4]. Several studies have suggested that fungal symbiosis helps plants to mitigate stress by increasing antioxidant activities [29,46,48].

Under salinity stress, phytohormones like ABA can protect plants by stomatal closure to minimize water loss and then mediates stress damage [49]. It is widely described that ABA contents in plants increase under salt stress [1,50]. However, our finding shows significantly lower ABA level in endophyte-associated plants as compared to endophyte-free plants. Previously, Jahromi et al. [51] observed the same findings after association of *Glomus intraradices *with lettuce plants. Similarly, when soybean were given salinity stress in the presence of phytohormones producing endophytic fungi (*Penicillium funiculosum *and *Aspergillus fumigatus*), ABA levels were declined [15,16], whilst the plants experienced lesser amount of stress. Since ABA is involved in the regulation of stress signalling during plant growth therefore, its biosynthesis can be affected by the presence of fungal interaction in abiotic stress. Although other studies suggests that fungal inoculation have increased the ABA content in leaves and roots compared with non-inoculation control plants [52]. However, the effect may fluctuate among difference class of microorganisms and plant species as some earlier reports have elaborated this [44,53]. There are several studied which narrates the same findings of low ABA levels under stress and fungal association [44]. Exogenous application of GA_3 _improved soybean salinity stress tolerance by increasing plant biomass while accumulating lesser ABA [54]. Iqbal and Ashraf [55] observed that GA_3 _application can results in altered level of ABA under salinity stress in *Triticum aestivum *L. Although, higher ABA in salinity is correlated with inhibition of leaf expansion and shoots development in different species [56] however, *P. formosus *inoculated plants counteracted adverse effects of stress by significantly increasing leaf area and shoot length as compared to control plants. Similarly, in case of cucumber plant's endogenous GAs, endophytic fungal application have rescued GAs biosynthesis as the level of bioactive GAs were much pronounced compared to sole NaCl treated plants. Phytohormones, like GAs have been widely known for their role in plant growth and various developmental processes during plant's life cycle [1,3,57]. Normal response of a plant to stress is to reduce growth by inter alia increasing ABA content and reducing GAs [56,57]. GA-deficient plants are more susceptible to stress than those with higher levels of this hormone [56]. The higher amount of GA_12 _in endophyte-treated plant under salinity stress elucidates the activation of GAs biosynthesis pathway, while higher production of GA_3 _and GA_4 _confirm plant growth maintenance during stress condition. Thus, by maintaining GAs and, therefore, growth under stressful conditions, the endophyte is having a detrimental effect on the plant long-term survival. There are many previous reports showing the ameliorative effects of exogenous application of GAs (GA_3_/GA_4_) and IAA on cucumber growth under abiotic stress [58-60], while very little or no information's are available on the regulation of plant endogenous hormones in association with phytohormones producing endophytic fungi under abiotic stress conditions. Some physiological evidence suggests that plants infected with endophytic fungi often have a distinct advantage against biotic and abiotic stress over their endophyte-free counterparts [61]. Beneficial features have been offered in infected plants; including drought acclimisation [62,63] enhanced tolerance to stressful factors such high salinity [12]. Foliar application of GAs has been known for its role in plant stem elongation and mitigation of abiotic stress [54-60] while the same was observed in current study that endophytes producing GAs triggered the adverse effect of salinity stress.

## Conclusion

*P. formosus *LHL10 produced many physiologically active and inactive GAs and IAA, which helped the *Waito-C *and Dongjin-byeo rice plants to grow well and significantly mitigated the negative impacts of salinity stress on cucumber plants. The *P. formosus *LHL10 also minimized the lethal effects of salt stress on cucumber leaf tissues as evidenced from EL, RWC, photosynthesis rate, nitrogen assimilation, antioxidant activity and lipid peroxidation. The cucumber plants inoculated with *P. formosus *LHL10 have ameliorated their growth by possessing lower levels of stress responsive endogenous ABA and elevated GAs contents. Current study reveals that such endophytic fungal interactions can improve the quality and productivity of economically important crop species. However, the favourable role of this fungus still needs to be investigated under field conditions.

## Authors' contributions

ALK undertook all the experimentation and manuscript preparation. MH and IJL participated in experimental design and supervision of the study while also participated in genomic DNA extraction and PCR analysis. SMK and YHK performed the GAs experiments while JHL and HYJ undertook microscopic analysis. All authors read and approved the manuscript.

## Supplementary Material

Additional file 1 **GC/MS-SIM analysis of HPLC fractions of pure culture filtrate of *P. formosus*. **The table contains retention times of various purified GAs through HPLC and GC/MS SIM data of GAs KRI values and ion numbers. Click here for file

Additional file 2 **GC/MS - SIM conditions used for analysis and quantification of the plant hormones**. The table contains GC/MS SIM conditions used for the detection of cucumber plant's endogenous GAs and ABA. Click here for file
