# Supplementary material for: Endophytic fungal association via gibberellins and indole acetic acid can improve plant growth under abiotic stress: an example of Paecilomyces formosus LHL10
Source: BMC Microbiol. 2012 Jan 12;12:3. doi: 10.1186/1471-2180-12-3 (PMC3268082; doi:10.1186/1471-2180-12-3)
Supplement: Additional file 1 — GC/MS-SIM analysis of HPLC fractions of pure culture filtrate of P. formosus. The table contains retention times of various purified GAs through HPLC and GC/MS SIM data of GAs KRI values and ion numbers. [file 1471-2180-12-3-S1.DOC]

**Additional file 1:**

**GC/MS-SIM analysis of HPLC fractions of pure culture filtrate of *P. formosus***

| **HPLC fraction No /RT** | **RT^a^** | **Sample/Standard** | **GAs^b^** | **KRI^c^** | ***m/z* (%, relative intensity of base peak)^d^** | | |
| --- | --- | --- | --- | --- | --- | --- | --- |
| 11~15 | 24.3 | sample | GA_1_ | 2674 | 506(100) | 491(13) | 313(17) |
|  |  | standard | [^2^H_2_] GA_1_ | 2674 | 508(100) | 493(15) | 315(19) |
| 11~15 | 25.48 | sample | GA_3_ | 2692 | 504(100) | 489(8) | 370(9) |
|  |  | standard | [^2^H_2_] GA_3_ | 2692 | 506(100) | 491(10) | 372(11) |
| 34~35 | 24.31 | sample | GA_4_ | 2506 | 284(100) | 225(80) | 289(70) |
|  |  | standard | [^2^H_2_] GA_4_ | 2506 | 286(100) | 227(76) | 291(71) |
| 7~9 | 24.67 | sample | GA_8_ | 2818 | 594(100) | 448(25) | 379(20) |
|  |  | standard | [^2^H_2_]GA_8_ | 2818 | 596(100) | 450(24) | 381(21) |
| 37~38 | 23.49 | sample | GA_9_ | 2305 | 298(100) | 270(78) | 227(48) |
|  |  | standard | [^2^H_2_] GA_9_ | 2305 | 300(100) | 272(77) | 229(48) |
| 41~43 | 22.21 | sample | GA_12_ | 2335 | 300(100) | 240(31) | 328(31) |
|  |  | standard | [^2^H_2_] GA_12_ | 2335 | 302(100) | 242(32) | 330(29) |
| 24~26 | 23.91 | sample | GA_20_ | 2485 | 418(100) | 375(45) | 403(14) |
|  |  | standard | [^2^H_2_] GA_20_ | 2485 | 420(100) | 377(45) | 405(13) |
| 36 | 25.03 | sample | GA_24_ | 2444 | 314(100) | 226(89) | 286(77) |
|  |  | standard | [^2^H_2_] GA_24_ | 2444 | 316(100) | 228(87) | 288(75) |

^a^RT – Retention time (in min); ^b^GAs – Gibberellins; ^c^KRI- Kovats retention indices; ^d^Identified as methyl ester trimethylsilyl ether derivatives by comparison with reference spectra and KRI data as elucidated by Gaskin and MacMillan (1991). Gibberellins are identified with three ions and quantified by first ion with comparison of labelled standards. About 50 μL of CF extract of CSH-6H results in various HPLC fractions.
