# Supplementary material for: Endophytic fungal association via gibberellins and indole acetic acid can improve plant growth under abiotic stress: an example of Paecilomyces formosus LHL10
Source: BMC Microbiol. 2012 Jan 12;12:3. doi: 10.1186/1471-2180-12-3 (PMC3268082; doi:10.1186/1471-2180-12-3)
Supplement: Additional file 2 — GC/MS - SIM conditions used for analysis and quantification of the plant hormones. The table contains GC/MS SIM conditions used for the detection of cucumber plant's endogenous GAs and ABA. [file 1471-2180-12-3-S2.DOC]

**Additional file 2:**

GC/MS – SIM conditions used for analysis and quantification of the plant hormones.

| Equipment | Hewlett-Packard 6890, 5973N Mass Selective Detector |
| --- | --- |
| Column | HP-1 capillary column (30m×0.25㎜ i.d. 0.25㎛ film thickness)  (J & W Scientific Co., Folsom, CA, USA) |
| Carrier gas | He (40 ㎖/min.); head pressure of 30 kPa |
| Source temperature | 250℃ |
| Oven conditions | GA : 60℃(1 min.) → 15℃/min. → 200℃(1 min.) →  5℃/min. → 285℃(5 min.) |
|  | ABA : 60℃(1 min.)→15℃/min.→200℃→5℃/min.→  250℃→10℃/min.→280℃ |
| Injector temperature | 200℃ |
| Ionizing voltage | 70 ev |
